# Effects of educational technology on reading achievement for Chinese K-12 English second language learners: A meta-analysis

**DOI:** 10.3389/fpsyg.2022.1025761

**Published:** 2022-11-07

**Authors:** Aohua Ni, Alan C. K. Cheung, Jieping Shi

**Affiliations:** ^1^Institute of Economics of Education, Faculty of Education, Peking University, Beijing, China; ^2^Department of Educational Administration and Policy, Faculty of Education, The Chinese University of Hong Kong, Shatin, Hong Kong SAR, China

**Keywords:** meta-analysis, English learning, educational technology, multimedia, reading, Greater China

## Abstract

No systematic published research has reviewed the impact of educational technology on English reading outcomes targeting the Chinese-speaking population. Therefore, this review intended to examine the impact of educational technology and its alternative types on reading achievement for Chinese English second language learners (ESLs) to understand how to best use technology applications to facilitate reading instruction. A total of 35 qualified studies were included in our analysis covering a sample size of 7,989 Chinese K-12 participants. Consistent with previous meta-analyses, our findings indicated a modest positive impact of educational technology on reading outcomes compared with the traditional teaching method (*d* = +0.37). For the five types of intervention identified in this review, we found that the comprehensive model had the largest impact (*d* = +0.60), followed by social media tools (*d* = +0.46), integrated online-learning system (*d* = +0.31), and multimedia-transmission model (*d* = +0.27). However, supplementary activities did not generate educationally meaningful effects on reading outcomes for Chinese ESLs (*d* = +0.05). The impacts of different moderators, implications, and limitations were also discussed. We argue for further integrating technology with the existing curriculum and pedagogy. The study adds to the second language (L2) reading literature corpus.

## Introduction

English second language learners (ESLs) constitute a growing part of the K-12 student population. In the era of globalization, the demand for English in countries such as China is growing rapidly. China boosts its economy through the opening-up policy and believes in the progressive role of English in promoting both individual success and the nation's competence (Jin and Cortazzi, 2002; Hu, 2005). One of the most substantial challenges for Chinese ESLs lies in reading. For example, despite substantial and sustained investments in curriculum reform over the past decades, Chinese ESLs were still reported to lag behind many of their Asian fellows in English reading, not to mention their counterparts in western countries (ETS, 2019).

Reading is argued to be the primary source of language input, particularly in the foreign language context, which may facilitate the acquisition of other language skills, accounting for much of the variability of the final learning outcomes (Qi, 2016). To master reading becomes even more critical considering the long-term effects of reading on children's personal development (Partanen and Siegel, 2014). It is widely believed that children who lack reading proficiency are more likely to be at high risk of being underachievers, dropping out of school, and developing behavioral and emotional disorders, which consequently will largely reduce their chances for college enrollment and future success (Daniel et al., 2006; Partanen and Siegel, 2014).

In response to the trend of technological integration in teaching and learning, a growing universal agreement has been reached on confirming educational technology as a practical solution to assist elementary and secondary school readers to succeed (Patel et al., 2018; Lee et al., 2020). For example, previous meta-analyses on the role of technology in reading have achieved unanimity, demonstrating that educational technology positively influenced K-12 students' reading accomplishment (Liao et al., 2007; Cheung and Slavin, 2012; Sung et al., 2015; Baye et al., 2019; Neitzel et al., 2022). The overall effect sizes of technology on reading achievement ranged from +0.05 to +0.83. However, most of these meta-analyses and their included studies were conducted in English-speaking countries, and the participants covered were first language (L1) learners instead of second language (L2) learners. In addition, some previous meta-analyses included studies with methodological deficiencies, such as a lack of a control group or a short duration (Kulik and Kulik's, 1991; Liao et al., 2007; Moran et al., 2008), highlighting the need to conduct a comprehensive meta-analysis with stringent criteria for ESLs in the Chinese context, which might help identify some common instructional model traits and trends across countries.

With the rapid development of modern technology and its extensive application in education, China seeks to popularize educational technology in K-12 classrooms. Chinese mainland began to reform instructional methods and promote educational technology nationwide in 2001 when the Ministry of Education issued the *Compendium of Curriculum Reform for Basic Education (Experimental)*, claiming to promote technology application in teaching and learning and propel intensive integration of multimedia technology with the discipline curriculum. In 2019, the State Council issued *China Education Modernization 2035*, pointing out that education informatization is the key to advancing the future growth of education. Taiwan started early computer application instruction experiments in the 1970s (Liao et al., 2007). It initiated a 5-year program: *National Program for e-learning*, in 2002 to stimulate research productivity in both academia and industry. In Hong Kong, the 1997 *Policy Address* and *Information Technology for Learning in a New Era Five-Year Strategy 1998/99 to 2002/03* indicated that Hong Kong attempted to develop an ideal model to make the best of educational technology in the new century. In 2014, the Education Bureau proposed the Fourth *Strategy on Information Technology in Education* to help students make the most of their educational opportunities by maximizing their potential in technology. In Macau, the Education and Youth Affairs Bureau has been focusing on promoting the development of educational technology in local schools. The Bureau funded the *Information Technology Education Plan* in 2005 and the *Education Development Fund* in 2007 to support and facilitate basic infrastructure construction, such as computers, networks, software, multimedia classrooms, and teaching platforms.

Regarding these actions, it is necessary to investigate the impact of technology implementation on Chinese ESLs' reading performance and, more importantly, to understand how to best use educational technology to facilitate reading instruction given the time, expenses, and resources restriction. The research questions are as follows: (1) Do educational technology applications enhance reading performance for Chinese K-12 ESLs? (2) What types of technology are most effective for Chinese ESLs? (3) How do other moderators affect the reading outcomes for Chinese ESLs?

The present meta-analysis is significant in the following ways. First, considering the effectiveness of educational technology in language learning, many Chinese schools have incorporated technology as an integral part of the English curriculum, particularly after the COVID-19 pandemic, which has led to the implementation of education technology reforms worldwide (Ni and Cheung, 2022). Following the challenges of the digital age, schools and teachers are now faced with the dilemma of determining which of the various types of technological interventions best support English instruction in K-12 classrooms. This meta-analysis intends to provide some implications for education policy and practice. Second, mastering reading is critical for Chinese ESLs considering the subject status of English and the long-term effects of reading on children's development. For L2 learners, reading development necessitates additional external interventions that provide greater input and lead to enhancements (Stephens, 1997). Defining the role of educational technology and its alternative types is beneficial to facilitate Chinese ESLs' reading development. Third, to the best of our knowledge, no systematic meta-analysis has reviewed the overall impact of educational technology and compared different instructional models on reading outcomes for Chinese K-12 ESLs. This paper intends to fill this gap and contribute to the increasingly recognized evidence-based education reform in Greater China (Slavin et al., 2021). The investigation of effective interventions will help to better support the technological advance in China, with implications for researchers and practitioners in developing countries.

## The technology debate

Whether and how technology affects learning accomplishment has led to considerable debate since the 1980s. In the “media effects” debate, Clark (1983) compared media to trucks bringing goods without nutritional value, which would not influence learning but merely increase expense. He further explained that instructional approaches and pedagogy, not technology, changed educational experiences. By contrast, Kozma (1994) argued that technology, as part of the existing environment, worked in harmony with multiple aspects to affect the learning process. Kozma (1994) believed in the actual impact of technology in classrooms and advocated expanding the capabilities of educational media.

With the massive usage of technology in education, researchers attempt to understand the complexity of educational technology in a larger context (Castañeda, 2019). Learning is believed to be a process entwining individuals, teachers, methods, and the environment (Carter, 1996). Rather than being misinterpreted as the best-mediated intervention, educational technology should be accompanied by appropriate instructional theories and designs (Shrock, 1994). As the effect of educational technology has been extensively empirically demonstrated, it is more practical and beneficial to address replication and the optimal application of technology in educational contexts (Cheung and Slavin, 2012).

Since the turn of the millennium, innovation in education has been a hot topic in China. In 2001, the eighth round of China's national curriculum reform claimed to propel innovative teaching and learning methods and the intensive integration of educational technology and instructional curriculum. This reform was the impetus for the emergence of evidence-based empirical research and the subsequent discussion on the nature and direction of educational technology development in China (Xie et al., 2018). A strong criticism asserted by some influential Chinese scholars leveled at the “technology-oriented” thinking mode (Wang and Xie, 2012; Zhou et al., 2014). They doubted the actual effect of “technology fever” when new applications were continuously adopted after the “failure” of the last one. The purpose of “use for use” tended to simply extoll technology in the teaching and learning process, ignoring exploration of further applicability and integration. In addition, according to Xiong and Wang (2015), the prevalence of theoretical and logical reasoning papers instead of empirical research limited the study of educational technology to the concept surface. Rather than denying the impact of educational technology, these arguments advocated careful considerations for technological development to shift from the teacher-centered transmission mode to the student-centered technology blending mode and strongly encouraged more evidence-based research (Wang and Xie, 2012; Zheng and Wu, 2013; Xiong and Wang, 2015). In light of this, performing a synthesis of experimental research to respond to the arguments and develop large-area applications is essential.

## Literature review

### Reading development

Reading is a complex process requiring various literacy skills to achieve proficiency. Panel (U.S.), N. R. (2000) identified five key elements of reading: phonemic awareness, phonics, vocabulary, fluency, and comprehension. For L1 readers, the emphasis of reading is put on its developmental characteristic as a reader's ability matures progressively over time to acquire necessary component skills to lead to mastery. Wolf and Stoodley (2008) described five stages of reading development. In stage 1 (e.g., preschool), readers are intended to acquire phonemic awareness and intuitive perception abilities. And in stage 2 (e.g., 6–7 years old), they are expected to establish word-sound relationships and develop initial vocabulary. In stage 3 (e.g., 7–9 years old), children start to decode words and chunks to increase fluency and preliminary understanding. Then they learn to read for learning and grow to be strategic readers to “synthesize information,” make inferences, and “repair faulty comprehension” (Wolf and Stoodley, 2008, p. 138) in stage 4 (e.g., 9–15 years old). In the last phase (e.g., 16 years old and above), readers can read across sources and disciplines to integrate conclusions and navigate multiple viewpoints. Proficient L1 readers are likely to master these skills rapidly within the stage period, while the story for L2 readers may be different.

L2 reading is distinct from L1 reading since L2 acquisition involves two different languages and social contexts. L2 readers fall behind L1 readers from the very beginning considering the grasp of vocabulary, sense of grammar, and different literacy backgrounds (Grabe, 1991). Theoretically, transfer effects from linguistic variations have been posited as the primary source of difficulties for L2 readers. Schemes of prior linguistic knowledge, such as orthographic structure, word order, syntactic and discourse, as well as the cultural preference in logic comprehension, may all contribute to interference and make L2 reading difficult (Grabe, 1991; Koda, 2007). When learning a second language, the transfer occurs when drawing on a set of available rules. Rather than basic knowledge and skills, the transfer is mostly an internalized correlational process in which fixed attitudes and patterns are transmitted (Koda, 2007). Consequently, inherent cultural identities are argued to potentially affect the use of English in L2 learning, which, however, was doubted as a cultural stereotype. Sufficient L2 input may foster bilingualism and facilitate the maturation of “transferred competencies” (Koda, 2007, p. 18). Thus, L2 reading may be a matter of proficiency, necessitating interventions that provide greater input and lead to improvements (Stephens, 1997).

### How technology might improve reading outcomes

Cognitive Theory of Multimedia Learning (CTML), proposed by Mayer (2005), provides the theoretical framework for the effective incorporation of educational technology and language learning instruction. CTML has three assumptions, “dual channels, limited capacity, and active processing” (Mayer, 2005, p. 33). Dual coding suggests that information is gained *via* both verbal and visual presentation systems. When computers are incorporated into reading instruction, the mixed modality of presentations combines powerfully to promote meaningful learning (Moreno and Mayer, 2007). According to eye movement and cognitive load theories, dual channels have minimal processing capacity since learners can only keep one piece of information in their working memory at a time. The adoption of technology, therefore, can assist learners in remembering more elements of structure or chunks in reading (Mayer and Moreno, 2002). Furthermore, effective learning depends on the active involvement of learners with cognitive processing. Technology-assisted education can benefit active learning in five aspects, “dialoguing, controlling, manipulating, searching and navigating” (Moreno and Mayer, 2007, p. 311), which may result in increased input, subject comprehension, and timely feedback, thereby providing learners with more flexibility and control.

Technology possibly provides a cogent explanation to improve reading outcomes (Svensson et al., 2021). In theory, targeted interventions, such as one-to-one or small-group tutoring, are often regarded as the most effective for fulfilling readers' individual needs (Cheung and Slavin, 2013a). However, in the L2 reading setting, the implementation of targeted tutoring can be particularly complicated considering the enormous number of ESLs and the sustained cost of time, resources, labor, and personnel training (Kunkel, 2015). Educational technology has been advocated to respond to these challenges in language learning (Brooks et al., 2006). Computers are stimulating and may concentrate attention when learning is viewed as entertainment, hence fostering cognitive growth (Liu et al., 2010). Using computers and other technologies, a large group of students may have flexible access to specialized teaching without over-reliance on tutors or rigid timetables (Fasting and Lyster, 2005; Macaruso and Rodman, 2009). Given the increased availability of computers and mobile devices, interventions can also be used effectively at home as supplemental mediation or in classrooms as an integral component of the core curriculum (Kunkel, 2015). To improve the effectiveness of educational technology, a more scientific and integrated system of technology use that adheres to instructional design principles is encouraged (Mayer, 2005; Moreno and Mayer, 2007).

### Models of educational technology

Educational technology here refers to a variety of devices or electronic applications that can be used in K-12 classrooms to facilitate the teaching process and enhance learning achievement (Cheung and Slavin, 2012, 2013b). The use of educational technology is based on the concept of constructivism, which emphasizes knowledge acquisition through active involvement, learning, and practical application (Jumaat et al., 2017). The process of learning is more student-centered and less objective and fixed, with more room for the learner to create their own knowledge as opposed to passively absorbing it (Xie et al., 2018). In this active learning environment, students are encouraged to take an active role in their education by making their own learning decisions and participating in various learning activities under the supervision of their instructors. Previous meta-analyses have identified various types of technology intervention that might facilitate active learning: computer-assisted instruction (Blok et al., 2002), tablet-based learning (Alqahtani, 2020), mobile-assisted learning (Tingir et al., 2017), innovative technology applications (Cheung and Slavin, 2013a), web-based learning (Liao et al., 2007), tutoring learning (Xu et al., 2019), cooperative learning (Sung et al., 2016), supplemental learning (Major et al., 2021), and comprehensive model (Cheung and Slavin, 2013a). This study identified five learning models from our included studies: the multimedia-transmission model, comprehensive model, supplementary activities, integrated online-learning systems, and social media tools.

#### Multimedia-transmission model

Multimedia-transmission model still belongs to the teacher-centered teaching and learning model. Teachers usually use computer-assisted multimedia instruction, such as pictures, music, and videos, to arouse curiosity and bring in the study topic. In this model, technology is often considered a supplemental tool to help deliver knowledge directly.

#### Comprehensive model

Comprehensive model is an integrated learning approach that incorporates technology into the core curriculum. For example, integrating computer or mobile-assisted instruction with non-technology-based classroom activities. In Zhang (2018) study, each class started with a 20-min vocabulary memorizing competition with digital dictionary apps and whiteboard assistance. The instructor then summarized, gave feedback, and conducted variation teaching. After class, students in the experimental class were divided into small groups to engage in online forum discussion and inquiry.

#### Supplementary activities

Supplementary activities usually provide extra instruction at the students' evaluated levels of needs (Cheung and Slavin, 2013a). Unlike the comprehensive model, which is integrated closely with the in-class instruction, supplementary activities such as *Destination Reading* are used as supplemental learning outside the classroom. For example, in Zhang (2007) study, students were offered one or two additional weekly training sessions to use the online learning software and resources to facilitate English reading.

#### The integrated online-learning system

The integrated online-learning system usually refers to the learning management systems or platforms that are highly functional and interactive. Students are encouraged to prepare, review, finish exercises, and interact with classmates and teachers *via* this system before and after class. Teachers can use the system to monitor learning progress and give feedback more efficiently.

#### Social media tools

Social media tools refer to the social media applications that facilitate learning, such as WeChat and Facebook. Social media tools provide a smoother, more direct communication tool between students and teachers. For example, Wu (2012) asked students to complete the individual assignment at first and then released the work online *via* Facebook. Then students read each one's work and gave comments.

### Previous meta-analyses and reviews

Over the past decades, dozens of reviews have been conducted to examine the impact of educational technology on reading achievement (Kulik et al., 1985; Liao et al., 2007; Cheung and Slavin, 2013a; Tingir et al., 2017; Baye et al., 2019; Neitzel et al., 2022). While the majority reported a similar positive conclusion on the effects of educational technology, the overall effect sizes in these meta-analyses varied largely from +0.05 to +0.83 (see Table 1). Although many research reviews have been available in the field, there are limited country-specific studies on reading interventions. In addition, the bulk of the included studies targeted L1 instead of L2 readers.

**Table 1:** Summary of major meta-analyses on effects of educational technology on reading achievement.

**References**	**Year covered**	**Educational level**	**Number of studies (reading related)**	**Effect size**
Kulik et al. (1985)	1966–1982	Elementary	7	0.42
Kulik and Kulik's (1991)	1974–1986	Kindergarten to secondary	18	0.25
Soe et al. (2000)	1982–1997	Elementary and secondary	17	0.13
Blok et al. (2002)	1990–2000	Kindergarten to elementary	42	0.19
Liao et al. (2007)	1990–2003	Elementary	11	0.83
Moran et al. (2008)	1988–2005	Secondary	20	0.49
Cheung and Slavin (2011)	1978–2010	Elementary and secondary	84	0.16
Cheung and Slavin (2013a)	1985–2011	Elementary (Struggling readers)	20	0.14
Sung et al. (2015)	1993–2013	Kindergarten to college	14	0.55
Sung et al. (2016)	Unspecified	Kindergarten to college	41	0.59
Tingir et al. (2017)	2011–2014	Elementary and secondary	3	0.67
Baye et al. (2019)	2009–2016	Secondary	23	0.11
Xu et al. (2019)	2005–2017	Elementary and secondary	19	0.60
Neitzel et al. (2022)	2004–2013	Elementary	4	0.05

It is worth mentioning that many reviews included studies with methodological deficiencies, such as no control group (e.g., Soe et al., 2000; Blok et al., 2002; Liao et al., 2007), inconsistent or brief duration (e.g., Kulik et al., 1985; Moran et al., 2008; Sung et al., 2015), lack of initial equality (e.g., Liao et al., 2007; Moran et al., 2008), non-standardized outcome measure (e.g., Soe et al., 2000) and teacher effect (e.g., Kulik and Kulik's, 1991). For example, in Kulik and Kulik's (1991) review, nearly one-third of the 53 studies lasted < 4 weeks. Short-duration studies are usually criticized for creating deceptive performance boosts since the treatments cannot be replicated over a longer school period (Cheung and Slavin, 2013a). Kulik and Kulik's (1991) also included studies without control for teacher effect, which would interfere with findings as the gains may be attributed to teachers instead of technologies. In the case of Liao et al. (2007), five selected studies were one group repeated measure without a control group design. Lacking a control group may discount research validity when there is no control for natural growth during the intervention process (Cheung and Slavin, 2016). Another point of Liao et al. (2007) is that the design of 30 included studies with no initial equivalence made it impossible to reach fairness and study causality between the control and treatment groups before the manipulation began. These methodological issues may help to explain the extensive variance and some of the large effect sizes.

Then Cheung and Slavin (2011) applied more consistent and stricter criteria to include studies that met high methodological standards. Eighty-four studies from 1978 to 2010 were identified with a total sample size of over 60,000 K-12 students. Findings indicated that educational technology had a small positive effect on reading performance with an overall effect size of +0.16. They also compared some intervention types and concluded that supplemental instruction approaches no longer yielded a promising impact. The rigorous inclusion criteria have been welcomed by many subsequent reviews in different fields (Cheung and Slavin, 2013a; Xie et al., 2018; Baye et al., 2019).

Recent meta-analyses (Baye et al., 2019; Neitzel et al., 2022) may raise the topic of whether educational technology is more successful than other reading treatments. These meta-analyses assessed the effects of various reading interventions on elementary and secondary school struggling readers. They found that the effect sizes of educational technology on reading outcomes at the primary and secondary levels were +0.05 and +0.11, respectively. The numbers were too small to produce educationally meaningful effects in reading. Besides, the effectiveness of educational technology did not outperform other intervention approaches. Programs using one-to-one tutoring, cooperative learning, and writing-focused strategies showed more positive results than technology programs. These results may alarm researchers to interpret the impact of educational technology with more caution and suggest a deeper integration of technology and instruction.

With advances in mobile networks and distance education, the past decade has witnessed increasingly innovative use of technology in the language domain. For example, Sung et al. (2015) found out of 14 studies that mobile-assisted instruction had a moderate effect of +0.55 on reading achievement. The results were congruent with those of Tingir et al. (2017), who discovered a similar relationship between mobile-related therapy and improved reading performance. In addition, Ma et al. (2014) investigated the effectiveness of a more intelligent tutoring system (ITS) on language learning. The system is highly interactive to monitor cognitive learning through the whole learning process, from the transmission to feedback and adaptivity. They reported an overall effect size of +0.34. Similarly, Xu et al. (2019) reviewed 19 studies and reported a moderate effect size of +0.60 for intelligent tutoring systems. Their findings underscored the necessity to determine the effectiveness of various technologies to best support English reading education in China.

## Methods

**Figure 1:**
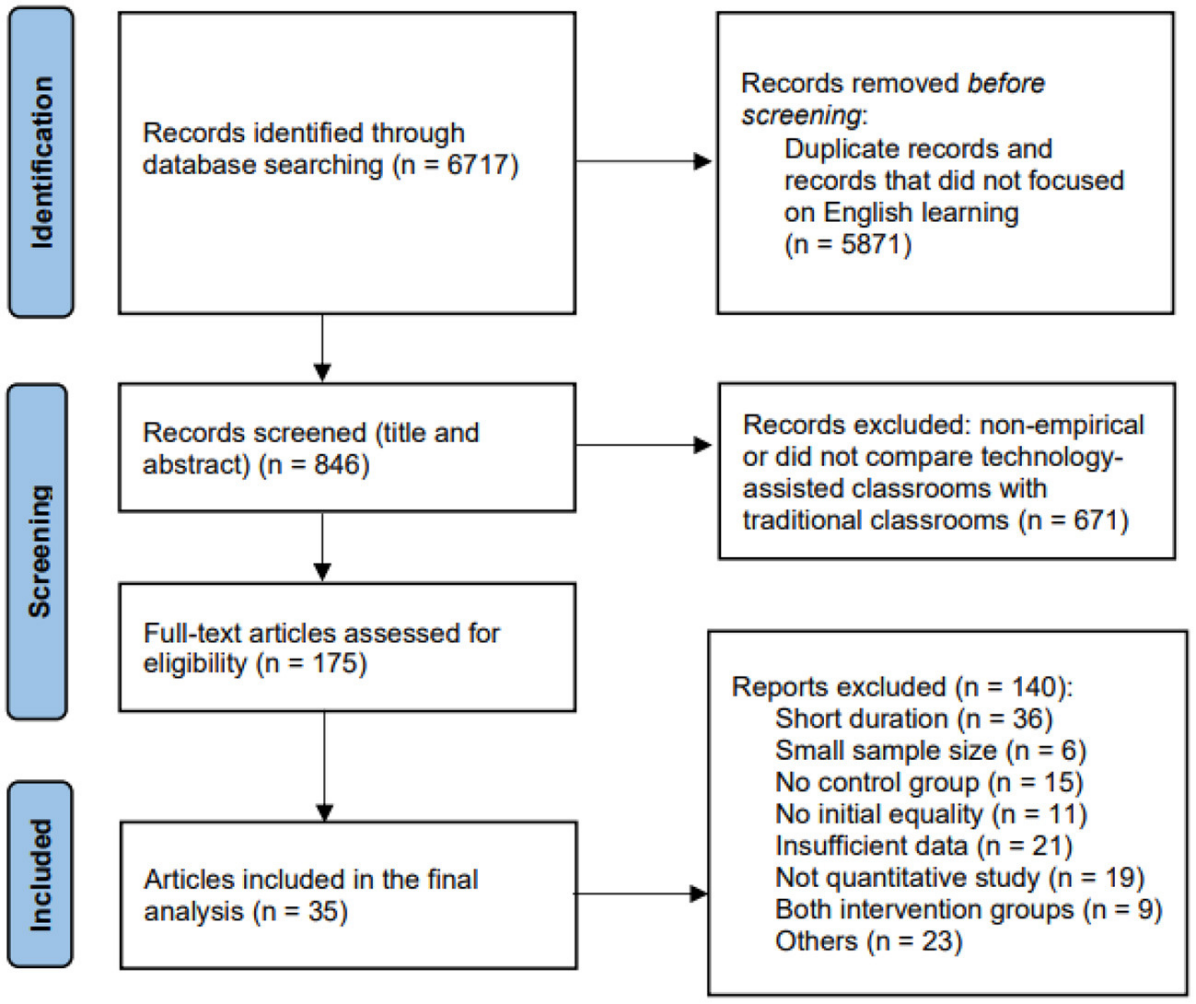
Flow diagram of literature search process.

This review employed the meta-analysis method proposed by Glass et al. (1981). This technique consists of five elements: (a) locate all potential studies; (b) screen studies using certain criteria; (c) code study data and features; (d) calculate effect sizes; and (e) run comprehensive statistical analyses. We followed the Preferred Reporting Items for Systematic and Meta-analysis (PRISMA) flowchart to select the included studies (see Figure 1).

### Literature search procedure

A literature search was conducted from the educational databases, including Web of Science, ERIC, JSTOR, and ProQuest, by creating the query (e.g., Reading^*^ AND (English^*^ OR ELL^*^) AND China^*^ AND (experiment^*^ OR intervention^*^ OR treatment^*^) AND (“educational technology” OR “instructional technology” OR “computer-assisted instruction” OR “multimedia” OR “mobile” OR “web-based”). We also retrieved studies from the major Chinese database: China National Knowledge Infrastructure (CNKI) (e.g., 英語 AND 實 閱讀 AND (實驗 OR 干預) AND (教育技術 OR 電腦 OR 手機 OR 多媒體 OR 網絡). If the search query did not work in some databases, we used the alternative combination of different keywords. We restricted the research to “all fields” between 2000 and 2020. In addition, we conducted comprehensive web-based searches from Google Scholar and publisher websites. References from previous reviews and qualified studies were also examined as supplementary. When including studies, we also added unpublished theses. There are two reasons why unpublished studies should be included. First, according to Cook et al. (1993), 78% of meta-analysis authors agreed that unpublished studies should be included in the reviews. When applying the same rigorous inclusion criteria, the combination of published and unpublished material would result in the most reliable synthesis of accessible data. Second, it is known that published studies have a greater likelihood of producing statistically significant results (Cheung and Slavin, 2016). The inclusion of unpublished data is a common way to avoid publication bias (Trespidi et al., 2011).

### Criteria for inclusion

We established the following criteria to include studies, referring to the reviews of Cheung and Slavin (2011, 2012, 2013a).

1. The studies assessed the impact of educational technology on 1st−12th grade English reading performance.
2. The studies should take place from 2000 to 2020 in the Greater China (Mainland, Taiwan, Hong Kong, and Macau) to examine the reading performance of Chinese ESLs.
3. The studies should employ a control group design and compare students in the treatment group using the technology-assisted learning approach and students in the control group taught by the traditional instruction method.
4. The participating students in each group (treatment or control) should be no < 30. There is no definitive answer to the question of how many units should be included in the control or treatment group based on previous research. We opted to use the criteria since a typical class in China is normally around 30 students.
5. The studies had to provide the pretest data unless using a randomized design of at least 30 units. The pretest difference between the two groups should be < 25% of a standard deviation to ensure baseline equivalence.
6. The duration of the study intervention was no < 12 weeks, representing the length of a regular school semester. We chose 12 weeks as the cutoff because we expected that the research could be replicated in a realistic setting. Due to novelty effects and high fidelity of implementation, previous research has revealed that a shorter period is more likely to produce greater impact sizes (Cheung and Slavin, 2016). Consequently, a number of meta-analyses have excluded trials with a length of < 12 weeks (Cheung and Slavin, 2013a,b).
7. The measuring tools of English reading outcomes in the studies should be quantitative.
8. The studies should report sufficient data to calculate the effect sizes.
9. To avoid confounding effects from teachers, each group should have at least two teachers. It is also acceptable if the two groups were taught by the same teacher.
10. Programs in the studies had to be replicable in a realistic school context.

### Coding

Many previous meta-analyses have found that methodological features influence the effect sizes of studies, such as research design, sample size, intensity, and intervention duration (Kulik and Kulik's, 1991; Cheung and Slavin, 2013b, 2016; Xie et al., 2018). In this study, two authors worked together to code the data to ensure data accuracy. The inter-rater reliability value exceeded 95%. The substantive features used in this review are sorted as follows.

1. Intervention types: multimedia-transmission model, comprehensive model, supplementary activity, integrated online-learning system, and social media tools
2. Research design: randomized, matched control.
3. Sample size: 60 ≤ N ≤ 99, 100 ≤ N ≤ 149, 150 ≤ N.
4. Publication type: published, unpublished.
5. Year: 2000–2010, 2011–2015, 2016–2020.
6. Intervention duration: ≤ 1 semester, 1 semester-1 school year.
7. Intervention intensity: ≤ 2 classes per week, >2 classes per week.
8. Grade level: elementary school (grade 1–6), secondary school (grade 7–12).
9. Region: towns, cities. We followed the administrative divisions of China to define special municipalities, provincial cities, and prefecture-level cities as “cities” and counties and villages as “towns”.
10. Post-test measures: teacher-made test, school final test, and professional test.

### Effect size calculation and statistical analyses

Effect size refers to the standardized difference between the mean for the control and treatment groups (Borenstein et al., 2021). If studies did not report effect sizes, we used other available statistical data like *p*-value, *F*, and *t* ratios to convert to Cohen's *d*. we also subtracted effect sizes for pretest from effect sizes for post-test if no adjusted means were reported. After calculating all effect sizes and variances, we employed Comprehensive Meta-Analysis (V3) for statistical analyses such as overall effect sizes, moderator analyses, and Q statistics.

## Results

### Mean effect size

#### Overall effects

**Figure 2:**
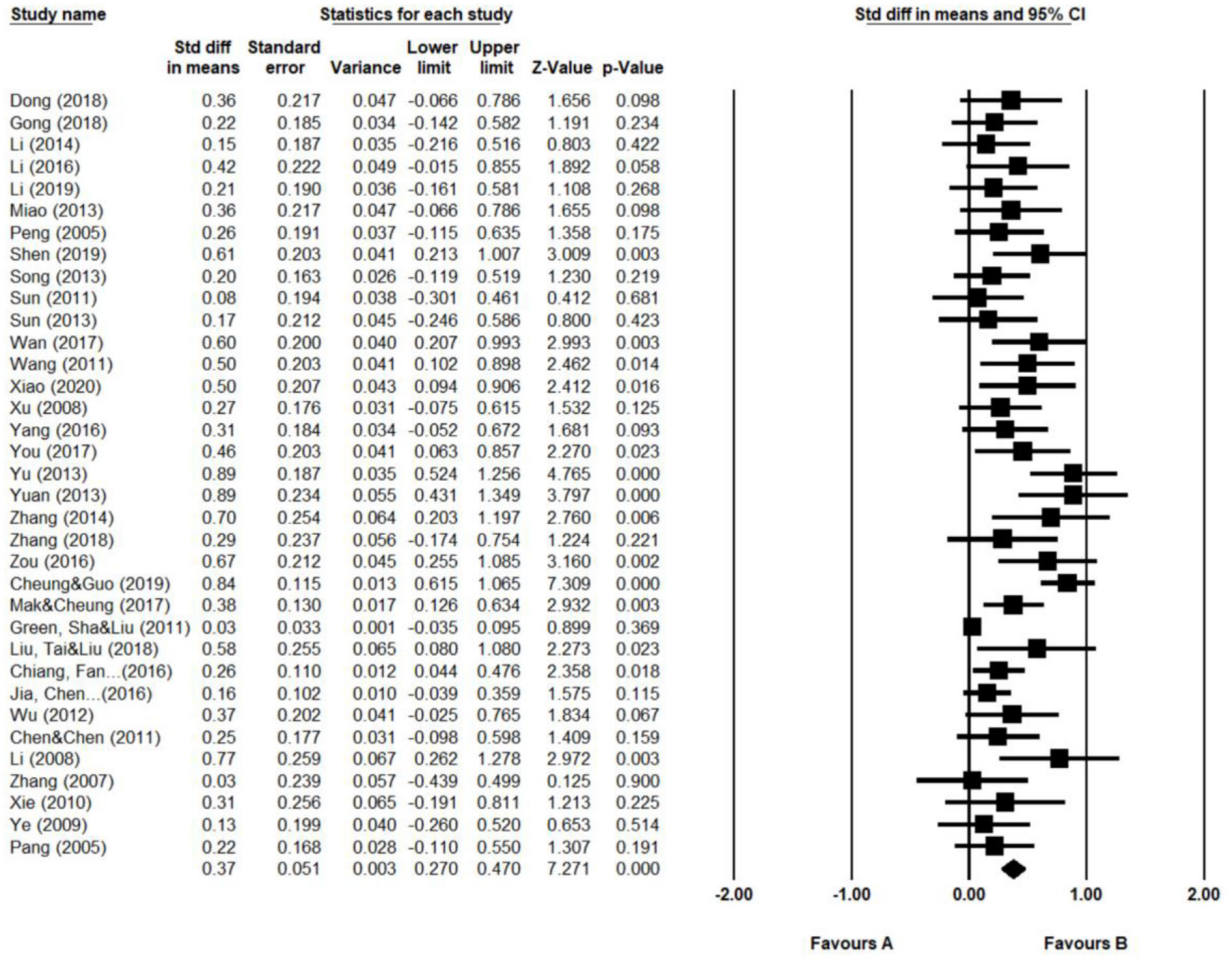
Forest plots of individual effect size concerning English achievement.

A total of 35 studies covering 7,989 K-12 participants have been included in this review. Figure 2 shows the forest plots of individual effect size. As indicated in Table 2, the overall mean effect size was +0.37. We chose to apply the result of the random-effects model because we assumed that the 35 studies were all heterogeneous in many features, such as intervention types, sample size, and grade level. Thus, it was not appropriate to attribute all the differences in effect sizes to the sampling error assumed by the fixed-effect model. The hypothesis was supported by the *Q*-test (*Q* = 115.90, *df* = 34, *p* < 0.00) and *Z*-test (*Z* = 6.51, *p* < 0.00), indicating that the true effect was indeed large than zero and the variance could be explained by more than simply sampling error. A random-effect model was then adopted.

**Table 2:** Overall effect sizes of FTC on English achievement.

	**Number of studies**	**Effect size**	**Standard error**	**95% confidence interval**		**Test of heterogeneity**		
				**Lower**	**Upper**	***Q*-value**	**df (Q)**	***p*-value**
1. Fixed	35	0.22	0.02	0.17	0.26	115.90	34	0.00
2. Random	35	0.37	0.05	0.27	0.47			

#### Sensitivity analysis

We used the one-study removal analysis in the sensitivity analysis to check for the existence of any possible outliers that might skew the effect sizes. We found that the range of effect sizes was still between the 95% confidence interval (0.27–0.47) after removing any one effect size. The removal would not affect the overall effect.

#### Publication bias

**Figure 3:**
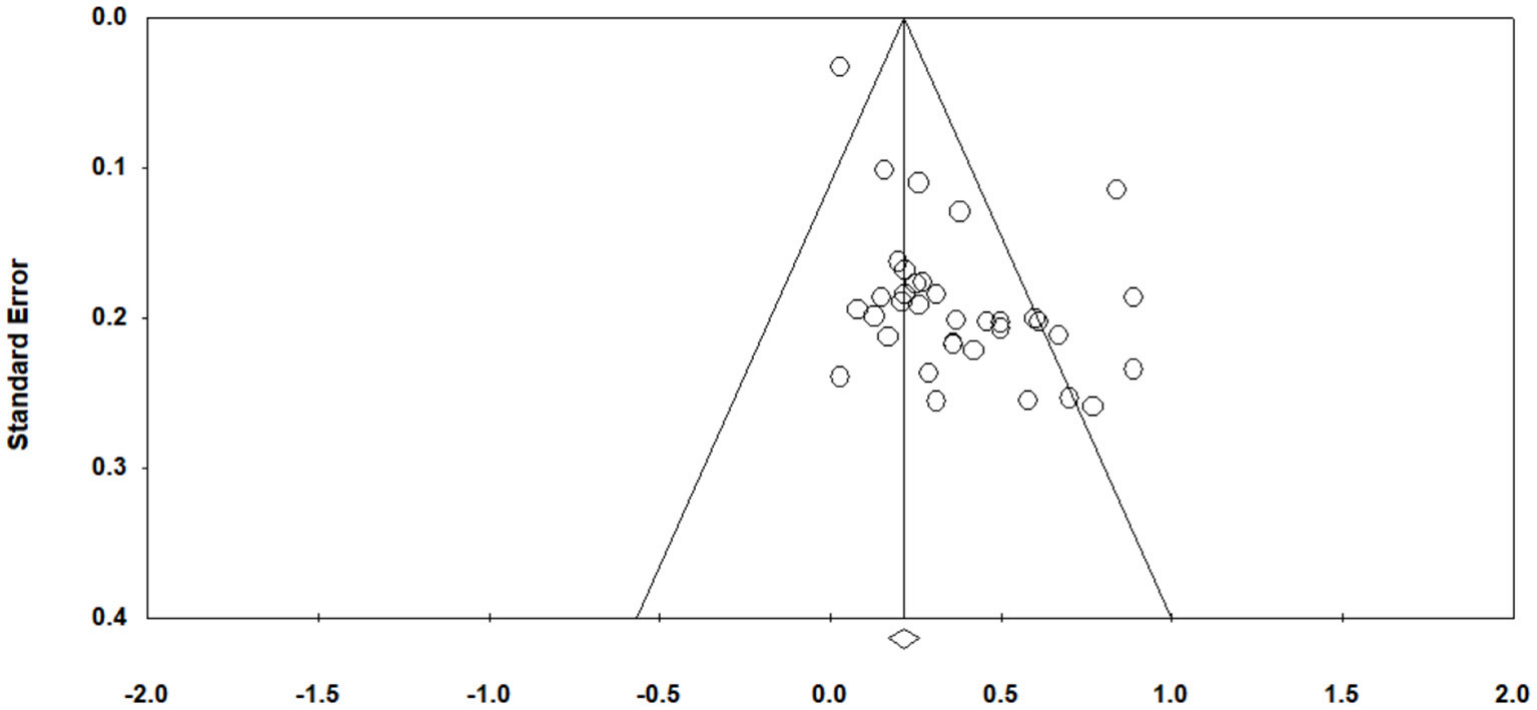
Funnel plot of standard error by effect size.

Published studies are more likely to report larger effect sizes and be included in meta-analyses (Cheung and Slavin, 2016). Then the question may be how much impact publication bias can have and whether the effects result from the bias. We used the common tests, Classic fail-safe *N* and Orwin's fail-safe *N* to check for publication bias (see Figure 3). The classic fail-safe *N*-test guided our estimation of how many additional studies would need to be collected and included in our analysis before the genuine overall effect became zero. The classic fail-safe *N-*test indicated that an extra number of 1,279 studies were needed in the analysis to invalidate the observed effect. In Orwin's fail-safe *N-*test, if 0.01 was defined as a trivial value, then 723 more studies needed to be added to the analysis to make the cumulative effect size non-significant. The results implied that publication bias could not entirely explain the observed positive effects, and we considered the overall effect size meaningful and robust.

### Moderator analysis

#### The effect sizes of different intervention types

Our review divided the studies into four categories: multimedia-transmission model (*N* = 11, comprehensive model (*N* = 8), supplementary activities (*N* = 5), integrated online-learning system (*N* = 5), and social media tools (*N* = 6). The effect size of comprehensive models was the largest (+0.60). Moreover, the effect size of studies using supplementary activities was the smallest (+0.05), which was too minimal to be educationally meaningful. Multimedia-transmission model studies produced the second smallest effect size (+0.27). The effect sizes of integrated online learning systems (+0.31) and social media tools (+0.46) were in the middle. We found a significant heterogeneous between-group effect (*Q* = 50.02, *df* = 4, *p* < 0.00). The heterogeneity showed that there existed variations in intervention effects in terms of the four different types of educational technologies.

#### Year

We divided the year of studies into three categories, 2000–2010 (*N* = 8), 2011–2015 (*N* = 11), and 2016–2020 (*N* = 16). The effect sizes for the three groups were +0.27, +0.36, and +0.42, respectively. Although the differences were not statistically significant (*Q* = 2.43, *df* = 2, *p* = 0.30), it seemed that research published in subsequent years had larger effect sizes.

#### Research design and sample size

A randomized trial (*N* = 2) is one in which students, classes, or schools were randomly allocated to conditions and the unit of analysis was at the random assignment level (Cheung and Slavin, 2011). By contrast, matched control studies (*N* = 33) tend to match the control and treatment groups on some key variables. The effect sizes for randomized and matched studies were +0.12 and +0.39, respectively. The effects showed a significant difference between the two designs (*Q* = 4.97, *df* = 1, *p* < 0.05). However, the results need to be interpreted with caution since there were only two randomized studies. For sample size, we divided studies into three categories: 60–99, 100–149, 150, and above. The effect sizes for the three groups were +0.46, +0.34, and +0.31, respectively. The heterogeneity test's results were not statistically significant (*Q* = 2.25, *df* = 2, *p* = 0.33).

#### Grade levels

As shown in Table 3, the mean effect size for elementary studies (*N* = 13, *d* = 0.40) was higher than for studies conducted in secondary schools (*N* = 22, *d* = +0.25). Nevertheless, the variance was not statistically significant (*Q* = 0.19, *df* = 1, *p* = 0.67).

**Table 3:** Subgroup analysis of FTC on English achievement.

**Study features**	**Number of studies**	**Effect size**	**Standard error**	**95% confidence interval**		**Test of heterogeneity**		
				**Lower**	**Upper**	***Q*-value**	**df (Q)**	***p*-value**
**Intervention type**	
Comprehensive model	8	0.60	0.10	0.39	0.80			
Integrated learning system	5	0.31	0.08	0.15	0.48			
Multimedia assisted learning	11	0.27	0.06	0.15	0.38			
Social media tools	6	0.46	0.08	0.30	0.62			
Supplementary activity	5	0.05	0.03	−0.02	0.11			
Total between	35					50.02	4	0.00***
**Year**	
2000–2010	8	0.27	0.07	0.13	0.41			
2011–2015	11	0.36	0.10	0.16	0.56			
2015–2020	16	0.42	0.06	0.30	0.54			
Total between	35					2.43	2	0.30
**Research design**	
Randomized	2	0.12	0.11	−0.10	0.34			
Match control	33	0.39	0.04	0.30	0.48			
Total between	35					4.97	1	0.03*
**Sample size**	
60–99	13	0.46	0.07	0.33	0.59			
100–149	16	0.34	0.05	0.24	0.45			
150 and above	6	0.31	0.13	0.06	0.55			
Total between	35					2.25	2	0.33
**Duration**	
≤ 1 term	20	0.35	0.05	0.26	0.44			
>1 term	15	0.38	0.09	0.21	0.55			
Total between	35					0.10	1	0.75
**Intensity**	
≤ 90 min	18	0.30	0.06	0.19	0.42			
>90 min	17	0.43	0.07	0.30	0.56			
Total between	35					1.93	1	0.17
**Grade**	
Elementary	13	0.40	0.07	0.25	0.54			
Secondary	22	0.35	0.06	0.23	0.48			
Total between	35					0.19	1	0.67
**Region**	
Town	10	0.37	0.12	0.13	0.62			
City	25	0.36	0.04	0.27	0.45			
Total between	35					0.01	1	0.92
**Post-test measures**	
Teacher made test	17	0.36	0.05	0.27	0.45			
School wide test	14	0.34	0.07	0.21	0.48			
Professional test	4	0.38	0.22	−0.05	0.82			
Total between	35					0.06	2	0.97

*p < 0.05.

**p < 0.01.

***p < 0.001.

#### Duration and intervention intensity

The length of one semester here was considered equal to around 4 months in the educational setting in China. Studies with a duration of ≤ 1 semester (*N* = 20) had an effect size of +0.35. The effect size of the other 15 studies, which had a duration of between 1 semester and a whole school year, was +0.38. No significant statistical difference was found between the two groups. Intervention intensity was divided into two categories: low intensity (≤ 2 classes per week) and high intensity (>2 classes per week). No heterogeneity was found between the two groups (*Q* = 1.93, *df* = 1, *p* = 0.17). The effect sizes for the 18 studies of low intensity and 17 studies of high intensity were +0.30 and +0.4, respectively.

#### Region and post-test measures

Regarding region, studies conducted in towns had an effect size of 0.37 (*N* = 10), while those conducted in cities had an effect size of 0.36 (*N* = 25). No statistically significant difference was found (*Q* = 0.01, *df* = 1, *p* = 0.92). In terms of post-test measures, the effect sizes for studies using teacher-made tests (*N* = 17), school final tests (*N* = 14), and professional tests (*N* = 4) (e.g., TOEFL) were +0.36, +0.34, and +0.38, respectively. No variance was found among the three categories (*Q* = 0.06, *df* = 2, *p* > 0.05).

### Meta-regression

We used meta-regression of the random-effects model to assess the relationship between multiple covariates and the technology intervention effect. In total, 71% of the between-study variance could be explained by the six covariates in the meta-regression model (see Table 4).

**Table 4:** Results of meta-regression.

**Random effects**	**Covariate**	**Coefficient**	**S.E**.	**95% CI**	***Z*-value**	***P*-value**
	Intercept	0.27*	0.12	(0.02, 0.51)	2.12	0.034
Design (MC)	R	0.00	0.11	(−0.21, 0.22)	0.05	0.961
Sample size (60–99)	100–149	−0.03	0.08	(−0.19, 0.14)	−0.31	0.760
	150 and above	−0.21*	0.09	(−0.39, −0.02)	−2.19	0.028
Intensity (≤ 90 min)	>90 min	0.08	0.08	(−0.09, 0.24)	0.84	0.399
Grade (Elementary)	Secondary	−0.03	0.07	(−0.17, 0.11)	−0.40	0.686
Region (Town)	City	−0.19*	0.08	(−0.34, −0.04)	−2.42	0.015
Intervention (Supplemental)	Comprehensive	0.54***	0.14	(0.27, 0.81)	3.92	0.000
	Integrated	0.37**	0.11	(0.14, 0.59)	3.23	0.001
	Multimedia	0.12	0.11	(−0.10, 0.34)	1.09	0.277
	Social media	0.43***	0.13	(0.18, 0.68)	3.39	0.001

*p < 0.05.

**p < 0.01.

***p < 0.001.

After controlling for other covariates, studies with larger sample sizes (150 and above) reported significantly smaller effect sizes than studies with small samples (60–99). In terms of region, in the subgroup analysis, no difference was found between studies conducted in towns or cities. However, in the meta-regression, results showed that studies conducted in cities had a smaller effect size of 0.19 than those undertaken in towns. Adding various covariates into the model might change the coefficients.

For intervention type, compared with supplementary activities, studies that used the comprehensive model, social media tools, or integrated online-learning system all showed significantly larger effect sizes. Meanwhile, the comprehensive model produced the largest impact on reading improvement, followed by social media tools. However, although the multimedia-transmission model showed a higher effect size than supplementary activities, the difference was insignificant. For other variables, no significant association was found between design, intensity, and grade level.

## Discussion

### Overall effect size

The present review included eligible studies to assess the impact of educational technology on reading performance in China. We found that educational technology produced a modest and positive effect size (+0.37). As compared to more conventional approaches, the use of educational technology in teaching does perform better in improving K-12 students' reading performance. The overall effect sizes from the previous meta-analyses ranged from +0.05 to +0.83 (e.g., Kulik et al., 1985; Sung et al., 2015; Neitzel et al., 2022). Concerning inclusion criteria, this review is most comparable to that of Cheung and Slavin (2011), which examined the impact of technology on reading achievement in English-speaking countries. Compared to their results (*d* = +0.16), the weighted average effect size reported in the present meta-analysis was somewhat larger. However, caution needs to be taken when extrapolating from our findings. First, only two of the studies were randomized studies. Moreover, a minority of the studies (17%) had a sample size of more than 150 participants. Previous reviews have revealed that the mean effect sizes for matched control studies were larger than those for randomized trials and that the mean effect sizes for small-scale studies were greater than those for large-scale studies (Cheung and Slavin, 2013b, 2016; Xie et al., 2020). In the meta-analysis conducted by Cheung and Slavin (2011), the proportions of randomized trials and large studies were 28 and 58%, respectively. This distinction may help explain why the effect size in the present review was larger than theirs. The findings underscore the need for more large-scale randomized research in the domain of educational technology in China.

### Effects of different technology models

One of the key findings in the present review was the varying effects of alternative types of educational technology on students' reading achievement. To determine which types of educational technology were most effective for Chinese ESLs, we classified the interventions in the 35 studies into five categories: the comprehensive model, social media tools, integrated online-learning systems, the multimedia transmission model, and supplementary activities (Liao et al., 2007; Cheung and Slavin, 2013b; Major et al., 2021). Our results indicated that, except for supplementary activities, all five technology models produced educationally significant impacts and might assist Chinese ESL students in achieving higher reading outcomes than the conventional teaching approach.

The mean effect sizes of the five technology types were different. For example, the comprehensive model had the largest effect (*d* = +0.60), which was 0.14 standard deviation larger than social media tools, 0.29 standard deviation larger than the integrated online-learning system, 0.33 standard deviation larger than the multimedia-transmission model, and 0.55 standard deviation larger than supplementary activities. According to Slavin (1991), a difference with an effect size of 0.25 is often deemed educationally meaningful. Thus, it might be extrapolated that the disparity between the comprehensive model and other types of technology was substantial enough to indicate learning enhancements. The difference indicated that the comprehensive model was demonstrably more effective than the other four models in English reading instruction. The results were consistent with the findings of Cheung and Slavin (2011, 2013a). This may be explained by the unique benefit of the comprehensive model that combines electronic and non-electronic activities in classrooms with the supplemental support of devices or software after class (Cheung and Slavin, 2011). The characteristics of high interactivity, intelligence, and integration endow a comprehensive model with a much greater impact on the reading outcomes for Chinese ESLs (Moreno and Mayer, 2007). Implementing a comprehensive model provides a promising way to incorporate educational technology into English reading instruction. Our findings suggest that a more integrated English reading classroom with careful instructional design and the use of technology may be more successful in enhancing the reading performance of Chinese ESLs.

Consistent with prior meta-analyses, we found that social media tools and integrated online-learning systems could improve students' reading performance (e.g., Ma et al., 2014; Sung et al., 2016; Xu et al., 2019). However, the findings of these prior reviews were too broad since they were more diverse and encompassed a variety of academic disciplines, educational levels, and study methodologies. The present meta-analysis narrowed its focus on English reading competency, included only elementary and secondary school students, and applied stricter criteria for experimental studies. Thus, our findings may add to the literature and provide a reference for the specific English reading research domain.

In recent years, social media tools such as WhatsApp and WeChat (*d* = 0.46) and integrated online-learning systems (*d* = 0.31) have gained popularity in English education and have great potential to increase reading performance. Two possible factors may help explain this. First, technological advances have radically transformed the educational landscape. Given the flexibility of offering instruction and assessment independent of time and place, the use of technology-based language learning has grown much more prevalent in the post-pandemic era in China (Ni and Cheung, 2022). Schools and teachers have been seeking ways to get instructional assistance from these technologies and to enhance the efficacy of technology-based English learning (Svensson et al., 2021). Second, using integrated online-learning systems and social media tools, students and teachers are able to remain in contact and engage in learning even while physically separated. With the potential for personal profiling, relationship-building, content generation, and socializing, these e-learning technologies enhance student engagement and make distance learning less remote (Lee et al., 2020; Major et al., 2021). For instance, “study with me” (Dong, 2018), an integrated learning management system that tightly connects teachers, students, and parents, was found to largely improve Chinese elementary students' English reading achievement (*d* = +0.34). For students, the system provides an abundance of English e-learning materials and can detect and correct errors in pronunciation and reading exercises. The system also makes it easier for English teachers to assign homework, give feedback, and communicate with parents online.

The multimedia-transmission model has its roots in the practice of Chinese English education (Xu, 2008; Wang, 2011). Multimedia-transmission model was a favored type of intervention in 11 studies, although its effect size was only +0.27. Given that transmission instructions have long been a focal point of China's conventional curriculum and pedagogy, the widespread adoption of this technology in English classrooms is not unexpected (Xie et al., 2018). Although the 2001 national curriculum reform encouraged a more student-centered constructivist approach to learning, transmission approaches were nevertheless appreciated and extensively implemented due to their cost-saving, knowledge-emphasizing, and skill-transmitting benefits (Wang and Xie, 2012). In most cases, however, computer-assisted multimedia was utilized as a tool to draw attention and transmit material, as opposed to being fully integrated with the curriculum and instruction (Xie et al., 2020). For example, Yang (2016) presented pictures or videos related to the unit theme at the beginning of each English reading class, followed by PowerPoint presentations of vocabulary, paragraphs, and grammar to boost students' attention and efficiency. However, reading is a comprehensive process entwining teachers' guidance, peer interaction, and environmental support (Cheung and Slavin, 2011). The evidence in the current review supports that the multimedia-transmission model should be updated with more advanced technology and further integrated with the English reading curriculum and pedagogy.

In contrast with the aforementioned intervention models, supplementary activities did not produce educationally meaningful effects on reading outcomes for Chinese ESLs (*d* = +0.05). The findings were consistent with previous reviews (Cheung and Slavin, 2011, 2012; Xie et al., 2020), revealing the minimal impact of supplementary programs on academic performance. Nonetheless, it would be premature to infer, without more research, that supplementary activities had the smallest effect on Chinese ESLs' reading achievement, given that only five studies were included in the subgroup. The findings support that technology is effective but is not a remedy (Cheung and Slavin, 2011). There is a need for systematic reflection on the nature of technology as well as more research into its integration with English pedagogy.

### Effects of study features

In addition to the primary findings, we intend to address some ramifications of the moderator analysis and meta-regression results. Previous research has revealed that studies with smaller sample sizes tended to produce larger effect sizes than those with large sample sizes (Slavin and Smith, 2009; Cheung and Slavin, 2016; Xie et al., 2020). In the present meta-analysis, we discovered that sample size was a critical factor that might influence the effect size of educational technology on Chinese ESLs' reading achievement. This may be related to the high fidelity of small-scale experiments. Generally, studies with smaller sample sizes are carried out with more consistency. Schools and teachers are more likely to support technology deployment and oversee its implementation process (Gu and Lau, 2021).

Previous studies have concluded that matched control studies with units of analysis normally at the student level tend to produce larger effect sizes than randomized studies (Cheung and Slavin, 2013b; Xie et al., 2018). The current meta-analysis confirmed their findings. Matched control studies may have a high degree of implementation and a greater potential to use self-developed outcome measures, both of which lead to higher intervention effects (Cheung and Slavin, 2016). However, the findings need to be interpreted with caution due to the limited number of randomized studies included in this review. There is an urgent need for more evidence-based randomized studies in China's English reading education.

The findings that region (degree of local economic development) was a significant variable were in accordance with previous reviews (Cheung and Slavin, 2013a; Xie et al., 2020). Our results indicated that technology interventions might be more effective when implemented at schools in towns than in cities. The findings suggested that students in disadvantaged areas need greater assistance with English reading from technology than their peers in developed regions (Xie et al., 2020). Compared with students in cities, students in lower economic developed towns are less likely to make use of educational technology applications when learning English reading (Cheung and Xin, 2019). These students are more likely to be motivated and focused while utilizing new technology applications for learning, resulting in improved implementation outcomes owing to novelty and interest.

Evidence in the present meta-analysis showed that studies published after 2015 tended to report larger intervention effects. The results were not surprising when considering the types of interventions used. For example, 8 out of 11 studies adopting the multimedia-transmission model were conducted between 2000 and 2014. Two of the five studies using supplemental activities were completed before 2010, while the other three were conducted between 2010 and 2015. Nevertheless, following 2015, studies tended to use more advanced interventions (e.g., learning management systems) and better integrate technology with instruction (e.g., the comprehensive model), resulting in a greater impact on Chinese ESLs' reading achievement (e.g., Jia et al., 2013; Liu et al., 2018; Zhang, 2018). The prevalence of technology-based instruction, notably after COVID-19, which has altered traditional teaching and learning, may also be a contributing factor (Ni and Cheung, 2022). Teachers and students are gaining expertise and confidence with the use of educational technology, which might result in more effective implementation.

The prior reviews concluded that study duration did not significantly affect learning outcomes (e.g., Liao et al., 2007; Cheung and Slavin, 2012; Sung et al., 2016). The evidence in this meta-analysis supported their findings. Meanwhile, it is worth mentioning that although the differences were not statistically significant, studies with short duration produced nearly tripled effect sizes than studies with high duration in this review. Short-duration studies usually result in larger effect sizes due to novelty and a high degree of implementation (Kulik et al., 1985; Kulik and Kulik's, 1991; Cheung and Slavin, 2016). Experimental studies need to last longer to be replicated in a real school setting. As with study duration, there was no significant difference in intervention intensity. More classes with technology did not lead to better English reading outcomes for Chinese ESLs. We may argue that implementation intensity is not an important moderator in educational interventions (Xie et al., 2018). However, we simply separated studies into two broad categories based on intensity. If future researchers intend to investigate this moderator further, they may need to establish more precise criteria to determine the effect of the time-variable factor.

## Limitations

There are some limitations to note in this meta-analysis. First, this meta-analysis only focused on Chinese K-12 ESLs and examined the impact of educational technology on English reading achievement. The findings cannot be generalized to preschool and higher education settings, nor can they provide information on academic outcomes in other domains. Second, the number of studies was insufficient in certain categories of the moderator analysis. For example, we only included two randomized studies. Among the five intervention types, we only included five studies for integrated online-learning systems and supplementary activities. The limited cases may reduce the accuracy of statistical power and limit the generalizability of the conclusion. Third, we argue for the credibility of measurements used in the studies, given that different schools developed various standardized tests. It is hard to know how the tests sampled reading. Therefore, the results need to be interpreted with particular caution. Fourth, this review only included experimental studies on reading achievement. However, insightful and valuable results from qualitative research are a cornerstone of the evidence-based education movement (Slavin et al., 2021). Future research may consider learning from qualitative studies to better understand how educational technology facilitates students' English reading.

## Conclusion and implications

This meta-analysis has revealed that educational technology has positive effects on Chinese ESLs' reading achievement. The use of educational technology has been of paramount importance in language education (Lee et al., 2020). Technology applications will undoubtedly continue to demonstrate their value in reading instruction. Therefore, the dilemma for schools and teachers is determining which interventions best support English reading in K-12 classrooms. Evidence in this review suggested that the comprehensive model, social media tools, integrated online learning system, and multimedia-transmission model could improve Chinese ESLs' reading outcomes. In addition, our findings indicated that the comprehensive model, social media tools, and the integrated online-learning system would be more effective than the multimedia transmission model and supplementary activities. Results imply that teachers are strongly encouraged to make thoughtful pedagogical designs and integrate the use of technology in terms of content and process to enhance the reading performance of Chinese ESLs.

Our review reveals some implications for researchers, educators, and policymakers. First, the findings respond to the technology debate in China (Zheng and Wu, 2013; Xiong and Wang, 2015) that educational technology does improve Chinese students' reading achievement and is worth implementation in K-12 English education. However, more attention should be paid to the types of interventions considering the varying effects of different types of educational technology on students' reading achievement. Schools and teachers should be knowledgeable about the effects and characteristics of various technologies in order to deploy them effectively in K-12 English classrooms. Second, our findings highlight the necessity for more large-scale randomized research in China concerning educational technology. For large-scale initiatives to be financed, governments at different levels should allocate funds to relevant projects. Third, it is critical to connect researchers, teachers, and school administrators. Researchers should be prepared to train frontline teachers and collaborate with schools and teachers to promote the implementation of technology applications. More frontline teachers and school administrators are encouraged to engage in the interventions. The experiences of these professionals are predicted to be a valuable addition to the existing studies.

## Data availability statement

The original contributions presented in the study are included in the article/supplementary material, further inquiries can be directed to the corresponding author.

## Author contributions

AN, AC, and JS engaged in literature search, screening, and coding. AN provided research idea, completed statistical analyses, and paper writing. AC organized and connected all parts of the paper. All authors contributed to the article and approved the submitted version.

## Conflict of interest

The authors declare that the research was conducted in the absence of any commercial or financial relationships that could be construed as a potential conflict of interest.

## Publisher's note

All claims expressed in this article are solely those of the authors and do not necessarily represent those of their affiliated organizations, or those of the publisher, the editors and the reviewers. Any product that may be evaluated in this article, or claim that may be made by its manufacturer, is not guaranteed or endorsed by the publisher.

## Appendix

**Table A1:** Coding table.

**References**	**Publication**	**Model**	**Design**	**Sample Size**	**Duration**	**Intensity**	**Level**	**Region**	**Measure**	**Effect Size**
Dong (2018)	U	IOS	M	86	1	Low	E	City	SWT	0.36
Gong (2018)	U	MTM	M	118	1	High	S	City	SWT	0.22
Li (2014)	U	MTM	M	115	1	Low	E	City	SWT	0.15
Li (2016)	U	MTM	M	83	1	High	S	City	TMT	0.42
Li (2019)	U	CM	M	112	2	High	S	City	TMT	0.21
Miao (2013)	U	MTM	M	86	1	High	S	City	TMT	0.36
Peng (2005)	U	MTM	M	110	2	High	S	Town	TMT	0.26
Shen (2019)	U	IOS	M	102	1	Low	S	City	SWT	0.61
Song (2013)	U	SMT	M	152	1	Low	S	City	SWT	0.20
Sun (2011)	U	MTM	M	106	2	High	S	City	SWT	0.08
Sun (2013)	U	S.A.	M	99	1	Low	S	City	SWT	0.17
Wan (2017)	U	SMT	M	104	1	Low	S	City	SWT	0.60
Wang (2011)	U	MTM	M	100	1	Low	S	Town	TMT	0.50
Xiao (2020)	U	CM	M	96	2	Low	E	City	SWT	0.50
Xu (2008)	U	MTM	M	130	2	Low	E	City	TMT	0.27
Yang (2016)	U	MTM	M	119	2	High	S	Town	TMT	0.31
You (2017)	U	SMT	M	100	2	Low	E	City	TMT	0.46
Yu (2013)	U	CM	M	126	2	High	S	City	SWT	0.89
Yuan (2013)	U	CM	M	80	1	High	S	City	TMT	0.89
Zhang (2016)	U	SMT	M	66	1	High	S	Town	SWT	0.70
Zhang (2018)	U	CM	M	72	1	High	S	City	PT	0.29
Zou (2016)	U	SMT	M	94	1	High	S	City	TMT	0.67
Cheung and Xin (2019)	P	CM	M	350	2	High	E	Town	PT	0.84
Mak et al. (2017)	P	CM	M	249	2	High	E	City	PT	0.38
Green et al. (2011)	P	S.A.	R	3640	2	Low	S	Town	PT	0.06
Liu et al. (2018)	P	IOS	M	64	2	Low	E	Town	TMT	0.58
Chiang et al. (2016)	P	IOS	R	374	1	Low	E	City	TMT	0.26
Jia et al. (2013)	P	IOS	M	389	2	Low	S	City	SWT	0.16
Wu (2012)	U	SMT	M	100	1	Low	E	City	TMT	0.37
Chen and Chen (2011)	P	S.A.	M	128	1	High	S	City	TMT	0.25
Li (2008)	U	CM	M	64	2	Low	E	City	TMT	0.77
Zhang (2007)	U	S.A.	M	70	1	Low	E	Town	SWT	0.03
Xie (2010)	U	MTM	M	62	1	High	S	Town	SWT	0.31
Ye (2009)	U	MTM	M	101	1	Low	E	City	TMT	0.13
Pang (2005)	U	S.A.	M	142	2	High	S	Town	TMT	0.22

U, unpublished; P, published; MTM, multimedia-transmission model; CM, comprehensive model; IOS, integrated online-learning system; SMT, social media tools; SA, supplemental activities; M, matched-control design; R, randomized experiment; 1, duration ≤ 1 semester; 2, 1 semester < duration ≤ 1 school year; Low, intensity ≤ 2 classes per week; high, >2 classes per week; E, elementary school; S, secondary school; TMT, teacher-made test; SWT, school-wide test; PT, professional test.
